# 16S rRNA Amplicon Profiling of Cranberry (*Vaccinium macrocarpon* Ait.) Flower and Berry Surfaces

**DOI:** 10.1128/MRA.01479-18

**Published:** 2019-01-24

**Authors:** Ghazal Ebadzadsahrai, Scott Soby

**Affiliations:** a Biomedical Sciences Program, College of Graduate Studies, Midwestern University, Glendale, Arizona, USA; b Biomedical Sciences Program, College of Graduate Studies and College of Veterinary Medicine, Midwestern University, Glendale, Arizona, USA; Indiana University, Bloomington

## Abstract

Bacterial populations associated with the surfaces of cranberry flowers and early fruits in wetlands bogs in Eastern Massachusetts were examined using pyrosequencing. The composition of bacterial populations was highly dependent on sample site, but the dominant phyla on both flower and berry surfaces were *Proteobacteria*, *Firmicutes*, *Actinobacteria,* and *Bacteroidetes.*

## ANNOUNCEMENT

Healthy plants host large and diverse populations of bacteria, but although soil and rhizosphere microbiomes have received a lot of attention, each plant organ has its own microbiome that is important for plant responses to environmental stimuli (1), plant growth promotion (2, 3), and suppression of infection (4). Cranberry (Vaccinium macrocarpon Ait.; Ericaceae) is a native North American plant and an important commercial crop in five U.S. states and two Canadian provinces (5); however, little is known about the populations of bacteria that inhabit the flowers and fruits of healthy plants.

As part of a combined culture-dependent and culture-independent study to establish a baseline for which types of bacteria were present in wild and cultivated cranberry plants and to determine their functional roles, cranberry flowers and fruits were aseptically removed from wild cranberry plants during early July during flowering/early fruit set at three independent sampling sights within the Cape Cod National Seashore in Massachusetts, and from a commercial bog maintained by the University of Massachusetts in Plymouth County, Massachusetts. Sterile cotton swabs moistened with 100 mM phosphate-buffered saline (PBS [pH 7.4]) were used to extract bacteria from 5 to 10 fruit or flower surfaces (6). The samples were suspended in PowerBead tubes and vortexed, and total DNA was extracted using a PowerSoil DNA kit (Mo Bio Laboratories). The 16S rRNA gene V4 region was amplified using the barcoded primer set 515f/806r (7), and 2 × 150-bp paired-end amplicon pyrosequencing was performed on an Illumina MiSeq platform, yielding a total of 77 Mbp. Paired-end reads were merged using PANDAseq, with correction for most errors (8), and then assigned to each sample by its unique barcode. Sequences were demultiplexed, barcoded primers were computationally removed, and the reads were filtered using default parameters in the QIIME pipeline (9). Following quality filtering, truncation, and chimera removal, operational taxonomic units (OTUs) were assigned by open-reference OTU picking (10) to sequences with 97% sequence similarity. A representative sequence was chosen for each OTU based on abundance, and taxonomic assignments were made using the RDP Classifier (11), with a confidence threshold of 80%.

Berry surfaces had more OTUs than did flowers at the same sampling site, as reflected by the Chao1 and Shannon diversity indices (Table 1), indicating that flower surfaces contribute to population composition on fruit surfaces, but additional bacterial inoculum comes from other sources. *Proteobacteria* was the dominant phylum on both flower and berry surfaces at each sampling site, primarily gammaproteobacteria (Table 1), followed by *Firmicutes*, *Actinobacteria,* and *Bacteroidetes*. *Tenericutes,* which includes the plant-pathogenic Phytoplasma spp., were observed in appreciable numbers only in flowers at a site where false blossom disease is present (Race Point). The most represented genera at all sites included Pseudomonas (24.71%), Burkholderia (15.69%), Acinetobacter (13.84%), and Sphingomonas (4.29%). For the first time, this study offers insight into the diversity of bacteria associated with the surfaces of the flowers and berries of Vaccinium macrocarpon.

**TABLE 1:** Summary of pyrosequencing analysis of cranberry flower and fruit surface bacteria in wild and cultivated bogs^*a*^

Analysis measure	Data by sample (SRA accession no.)							
	HH 1 flower (SRR7784148)	HH 3 flower (SRR7784150)	RP flower (SRR7784152)	SB flower (SRR7784154)	HH 1 berry (SRR7784147)	HH 3 berry (SRR7784149)	RP berry (SRR7784151)	SB berry (SRR7784153)
Diversity								
Estimated sample coverage (Good’s coverage)	0.9	1	0.99	0.99	0.97	1	0.98	0.99
No. of observed OTUs	62	21	38	88	67	254	307	191
Shannon diversity	4.26	0.17	2.28	1.72	3.4	5.93	2.74	2.81
Chao1 estimate	85.63	73.5	48.5	141.12	92.38	255.45	1,022	395.13
Counts/sample	27,103	45,558	13,638	16,088	44,262	9,776	217,975	22,127
Relative abundances (%) of bacterial classes								
*Alphaproteobacteria*	6.90	0.30	2.40	0.80	14.00	5.20	10.40	16.80
*Betaproteobacteria*	2.50		45.70	70.90	7.60	1.40		2.10
Deltaproteobacteria	0.70		0.20		0.70	0.50		
*Gammaproteobacteria*	49.60	98.60	14.10	25.80	55.20	23.50	75.70	62.80
*Bacilli*	18.50		0.50			1.50		0.20
*Actinobacteria*	15.20		0.70	2.20	17.20	3.30	4.50	12.90
*Bacteroidia*	2.50		1.00		2.20	25.80	8.90	0.20
*Anaerolineae*	1.40		0.20		0.10			
*Clostridia*	1.40		0.60		1.90	34.30		0.10
*Sphingobacteriia*	0.70							4.70
*Gemm-3*	0.40							
*Acidobacteriia*					0.40	0.70		
*Rubrobacteria*						0.10	0.10	
*Armatimonadia*						0.10		
*Cytophagia*						0.20		
*Flavobacteriia*			0.10					
*Erysipelotrichia*					0.60	0.80		0.10
*Mollicutes*			34.00			0.40		
*Verrucomicrobiae*						1.70		
*Deinococci*							0.10	
Other		1.00	0.10			0.10		

a The table includes population diversity measures and relative percent abundance of bacteria at the class taxonomic level expressed. HH indicates samples taken from the High Head sites, RP indicates samples taken from the Race Point site, and SB indicates samples taken from the UMass State Bog. The sample with the lowest number of samples (9,776) was chosen for the rarefaction level and for normalization. Bacterial taxa present at relative abundances of <0.1% were removed for clarity.

### Data availability.

Sequences were submitted to the NCBI database in the Sequence Read Archive under accession numbers SRR7784147, SRR7784148, SRR7784149, SRR7784150, SRR7784151, SRR7784152, SRR7784153, and SRR7784154.
